# Development and viability of biofilms grown on experimental abutments mimicking dental implants: An *in vivo* model

**DOI:** 10.4317/medoral.22868

**Published:** 2019-06-25

**Authors:** Berta Cortés-Acha, Rui Figueiredo, Vanessa Blanc, Agnès Soler-Ollé, Ruben León, Eduard Valmaseda-Castellón

**Affiliations:** 1 DDS, MS. PhD. Faculty of Medicine and Health Sciences of the University of Barcelona (Spain); 2 DDS, MS, PhD. Adjunct professor. Oral Surgery and Implantology Department, Faculty of Medicine and Health Sciences of the University of Barcelona. Researcher at the IDIBELL Institute, Barcelona (Spain); 3 MS, PhD. Microbiology Department Director. Dentaid Research Center, Cerdanyola del Vallés, Barcelona (Cerdanyola del Vallés, Spain); 4 MS. Microbiology Department Researcher. Dentaid (Cerdanyola del Vallés, Spain); 5 MS, PhD. R&D Manager. Dentaid Research Center, Cerdanyola de Vallés, Barcelona (Cerdanyola del Vallés, Spain); 6 MS, PhD. Associate professor. Oral Surgery and Implantology Department, Faculty of Medicine and Health Sciences of the University of Barcelona. Researcher at the IDIBELL Institute, Barcelona (Spain)

## Abstract

**Background:**

To determine whether an experimental abutment mimicking the macro- and microstructure of a dental implant is a suitable method for recovering biofilm, and to describe the features of biofilms formed around such abutments on healthy implants.

**Material and Methods:**

Experimental abutments were used in 15 patients without peri-implant diseases. After 14 days’ absence of dental hygiene in this area, the abutments were retrieved and analyzed through confocal laser scanning microscopy and scanning electron microscopy. The biofilm formation on the surface of the first 5 abutments was determined by a fluorescence-staining method using SYTO9 nucleic acid stain. In order to study the biofilm’s coverage and vitality, 10 additional abutments were assessed using live & dead bacterial viability. Descriptive and bivariate analyses of the data were performed.

**Results:**

A global plaque coverage of the abutments was observed in all cases. The submucosal area of the abutment was mostly covered with biofilm (over 21%). Moreover, significant differences between supra- and subgingival locations were detected.

**Conclusions:**

This *in vivo* experimental model allows detailed observation of the extensive plaque growth found on exposed experimental abutments mimicking dental implants when hygiene measures are absent. The biofilm coverage is significantly higher in the supragingival zone than in the subgingival portion.

** Key words:**Dental implants, biofilm, peri-implant diseases.

## Introduction

Since Brånemark defined osseointegration in the mid-1960s (1), oral rehabilitation has changed dramatically due to the introduction of dental implants. Although implant survival rates are generally very high (2), the prevalence of long-term biological complications is considerable (3,4). Peri-implantitis has been defined by some authors as an infectious chronic disease that affects osseointegrated functional implants (5), in which colonizing bacteria forming a biofilm seem to play a very important role (6). Other variables like a history of periodontal diseases, poor oral hygiene or smoking habits also influence the progress of this complication (7).

A dental biofilm has been described as a microbial community grown on teeth surfaces or any other hard non-shedding material. Immediately following the immersion of a hard surface into the oral cavity fluids, adsorption of macromolecules leads to the formation of a conditioning film, also known as acquired pellicle. This is formed by a variety of salivary glycoproteins (mucins, phosphoproteins, proline-rich and histidine-rich proteins), enzymes and other molecules that alter the charge and free energy of the surface, facilitating bacterial adhesion (8). After early colonizers (i.e. streptococcal and actinomyces species) have bound to the pellicle, the adhesion of secondary microorganisms takes place and the structure of the biofilm becomes thicker. Thick biofilms are characterized by poor oxygen diffusion in the deeper layers, which leads to the formation of a completely anaerobic environment. At this stage, the third set of colonizers — considered periodontopathogenic oral microorganisms — becomes established.

When bone loss occurs due to either remodelling or inflammation (e.g. peri-implantitis), the implant surface becomes exposed to the oral cavity and, as mentioned previously, salivary biopolymers attach to the implant surface. This can eventually lead to the formation of a pathogenic biofilm (9-13). The macro- and microstructure of the dental implants (presence of threads and rough surfaces) probably enhance this attachment, favoring peri-implant tissue inflammation.

**Figure 1:**
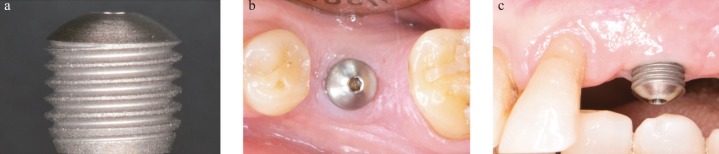
The experimental abutment. a) Biofilm collector abutment; b) Occlusal view of the abutment placed in one patient; c) Buccal view of the abutment placed in another patient.

Most of the available data on bacterial colonization and biofilm formation in dental implants are based on the analysis of titanium discs placed in splints (14-17). In the opinion of the present authors, this method has important limitations, since it does not reproduce a real clinical situation. Indeed, the biofilm samples collected with titanium discs only recover supra-mucosal biofilm, mainly composed of aerobic and microaerophilic microorganisms. This drawback is especially relevant since the subgingival area presents a favorable environment for the growth of more pathological bacteria. Other authors have proposed using removed failed implants, since this makes it possible to study the biofilm’s structure (18,19). However, it does not allow the initial phase of bacterial colonization to be analyzed. Finally, some reports have used healing abutments with different degrees of roughness to study biofilm formation (9,20,21). Nevertheless, these abutments did not reproduce the geometry and surfaces of dental implants. In the present authors’ opinion, the surface roughness (microstructure) and threads (macrostructure) of the implants are very important variables since they probably enhance plaque formation and complicate dental hygiene. Therefore, an experimental model that uses removable abutments which replicate exposed dental implants (with their supra and subgingival areas) is of great interest, since it would allow intact biofilms formed over the implants to be recovered. Moreover, this procedure is non-invasive and can be used in implants with or without a pathological condition (Fig. 1).

The main aim of the present work was to evaluate whether a custom-made abutment with the same macro- and microstructure as a dental implant is a suitable method for recovering biofilm; and, secondarily, to analyze the features of a biofilm formed on healthy dental implants after 14 days without oral hygiene measures. The main hypothesis of the present study was that custom-made removable abutments that simulate the structure of dental implants can be used to recover intact oral biofilm.

## Material and Methods

A non-randomized experimental study was conducted in a total of 15 subjects that had at least one dental implant placed in the Dental Hospital of the University of Barcelona (Spain).

The CONSORT statement guidelines (22) were used as a reference to report this study, which complied with the Helsinki declaration. The protocol was submitted to and approved by the Ethical Committee for Clinical Research (CEIC) of the Dental Hospital of the University of Barcelona (registered number 488) and all the patients signed an informed consent form before enrollment.

The inclusion/exclusion criteria and the description of the abutments used have been defined in a previous publication (23). Briefly, patients were excluded if any antibiotics or antiseptic mouthwashes were used during the study or in the previous 30 days, and the selected subjects were instructed not to use any oral hygiene measure over the abutment area. The participants could brush or floss the rest of the implants or teeth, but without using toothpaste. Once the abutment was placed (Fig. 1), the buccal area was marked using a diamond bur, and the number of exposed threads was recorded for later analysis. Participants with active periodontal disease were excluded. Periodontal patients were only included if the disease was considered under control, with a pocket probing depth (PPD) of ≤4mm and no bleeding on probing (BOP) at over 30% of the sites.

After 14 days, the abutment was removed and sent in a sterile snap tube with saliva at 4ºC to the microbiology facilities of the Dentaid Research Center (Dentaid SL, Cerdanyola del Vallés, Barcelona, Spain). The abutment was screwed to an implant analogue inside the snap tube. This was done to avoid the abutment’s touching any surface, thus avoiding biofilm disruption. The biofilm formation on the surface of the first 5 abutments was determined by a fluorescence-staining method using SYTO9 nucleic acid stain (Molecular Probes, Eugene, OR, USA). The abutments were immersed in 0.02 mM SYTO9 for 10 minutes at room temperature, avoiding exposure to light, then they were rinsed once with phosphate-buffered saline (PBS) and were examined under 10X magnification, using a Leica TCS SP5 confocal laser scanning microscope (Leica Microsystems, Heidelberg, Germany). The white laser was set at 482 nm, with emission bands between 500 and 540 nm.

Afterwards, in order to study the biofilm’s coverage and vitality (meaning the proportion of live to dead cells in the whole biomass), 10 additional abutments were assessed using the LIVE & DEAD Baclight Bacterial Viability Kit, L7012 (Molecular Probes, Eugene, OR, USA). The abutments were immersed in equal volumes of SYTO9 (0.02 mM) dye and propidium iodide (PI) (0.12 mM) dye diluted in PBS, and the mixture was incubated under the aforementioned conditions. The white laser was set at 482 nm for SYTO9 and at 514 nm for propidium iodide, and the emission bands were 500 to 540 nm and 570 to 700 nm, respectively. Two sides (buccal and palatal/lingual) and three fields per side (supragingival, intermediate and subgingival) were observed under 10X magnification.

The biofilm area was quantified using MetaMorph® v1.5 software (Molecular Devices, LLC, Sunnyvale, USA). Five regions of interest (ROI), always selected in the same relative position, were quantified using the maximum projections obtained from each field. The 2D and 3D reconstruction was performed using Imaris® v.7.1 software (Bitplane AG, Badenerstrasse, Zurich, Switzerland).

Afterwards, all the abutments were fixed in a 3.5% formaldehyde solution, vacuum-dried and observed through the scanning electron microscope (SEM; Merlin FE-SEM®, Carl Zeiss, Oberkochen, Germany) at 56X and 100X.

-Statistical Analysis

The data were processed using IBM SPSS 22.0 software (IBM, New York, USA). Since the sample size was limited, the median and interquartile range (IQR) were calculated for all the scale variables. All the ROI had the same area. The total area of the ROI that was covered with biofilm was calculated. The Wilcoxon signed rank test for paired data was used to compare the coverage of the surface of the abutment (supragingival vs. subgingival, buccal vs. lingual and live vs. dead cells). Differences in coverage between patients with and without a history of periodontal disease were assessed with the Mann-Whitney U test. Results were considered statistically significant when *p*<0.05.

## Results

The first 5 abutments stained with SYTO9 and observed through CLSM were retrieved from 3 men and 2 women with a median age of 59 (IQR=13,5 years). A biofilm covering the entire surface of the abutments was observed. SEM disclosed a thick biofilm with high coverage in the supragingival portion of the abutment. The biomass covering the abutments included bacteria and also epithelial cells, especially in the subgingival portion (Fig. 2.1).

**Figure 2:**
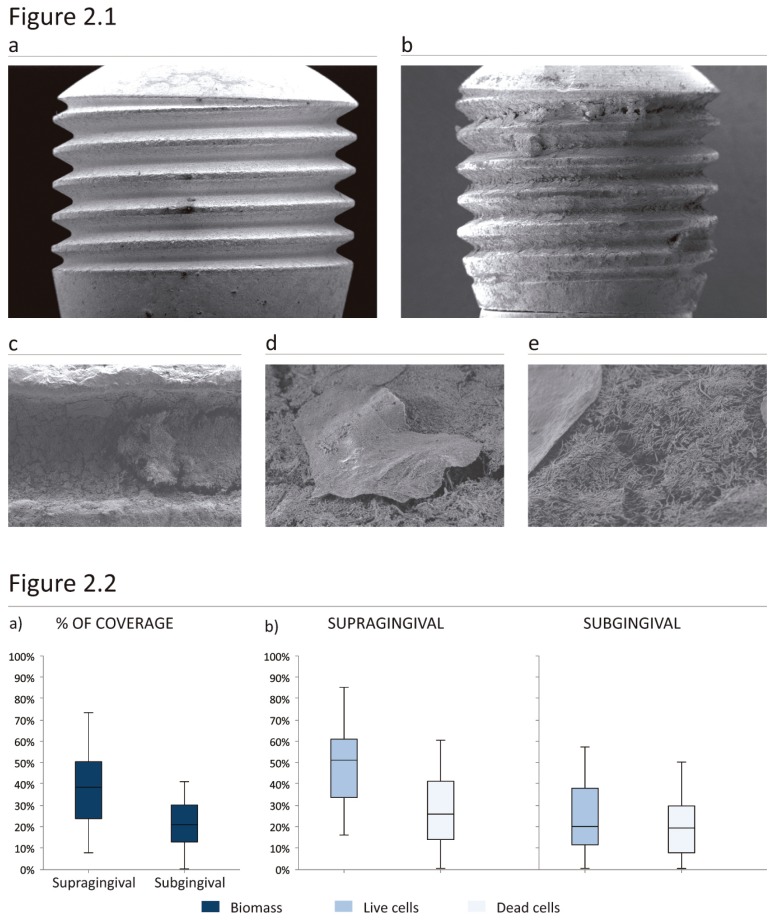
1: (A) Unused abutment, magnification 18 X, (B) Abutment after study period, magnification 18 X, (C) Biofilm formed in the concavity of the thread, magnification 1.00K X, (E) Epithelial cell attached to the biofilm, magnification 1.41K X, (D) biofilm underneath an epithelial cell 5.06K X. 2: Coverage and vitality by location (supragingival and subgingival). % coverage is the percentage of the total surface area occupied by bacteria on the ten abutments, Vitality is the percentage of live cells (blue) and dead cells (light blue) in the total biomass.

Figure 2.2 shows the results of the 10 abutments that were assessed for vitality (meaning the proportion of live to dead cells in the whole biomass). The abutments were placed in 10 non-smoking women with a median age of 59 years (IQR=15 years). Five subjects were periodontally healthy and 5 had a history of periodontitis.  Table 1 summarizes the main clinical features of the participants.

**Table 1:**
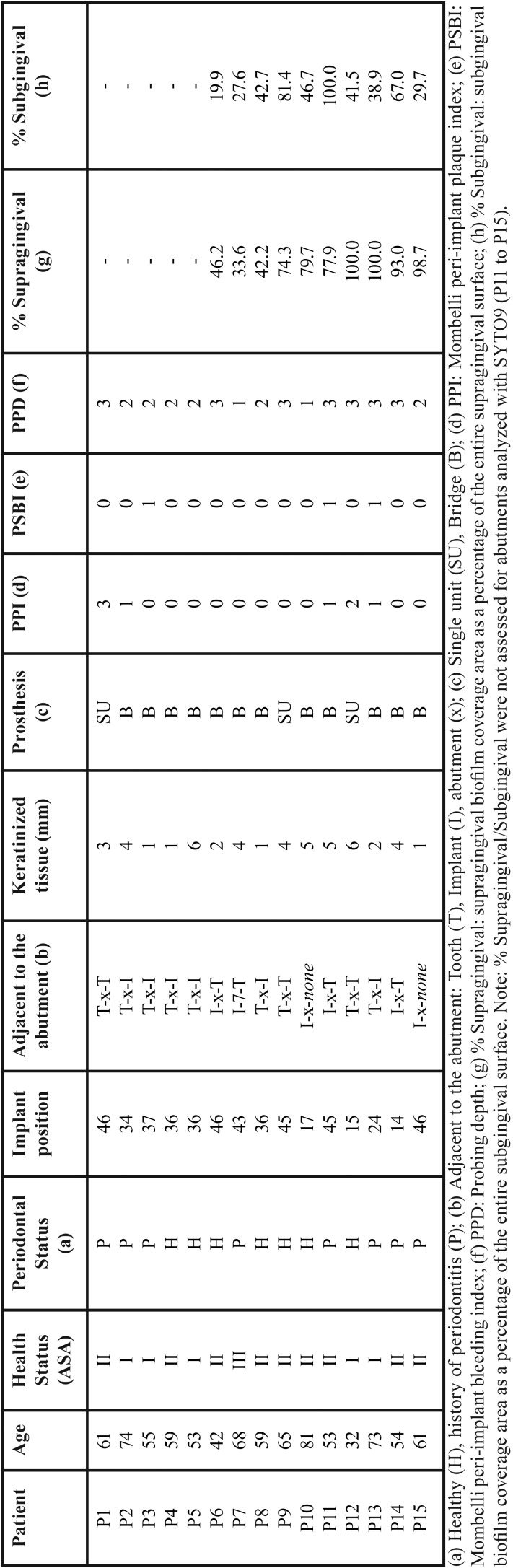
Main clinical features of the patients included in the study.

Thirty-eight percent of the supragingival surface of the abutments and 21% of their subgingival area were covered with biofilm (*p*= 0.013) (Fig. 2.2a). In absolute numbers also, the coverage in the supragingival portions was greater for both live (*p*= 0.047) and dead bacteria (*p*= 0.028).

**Figure 3:**
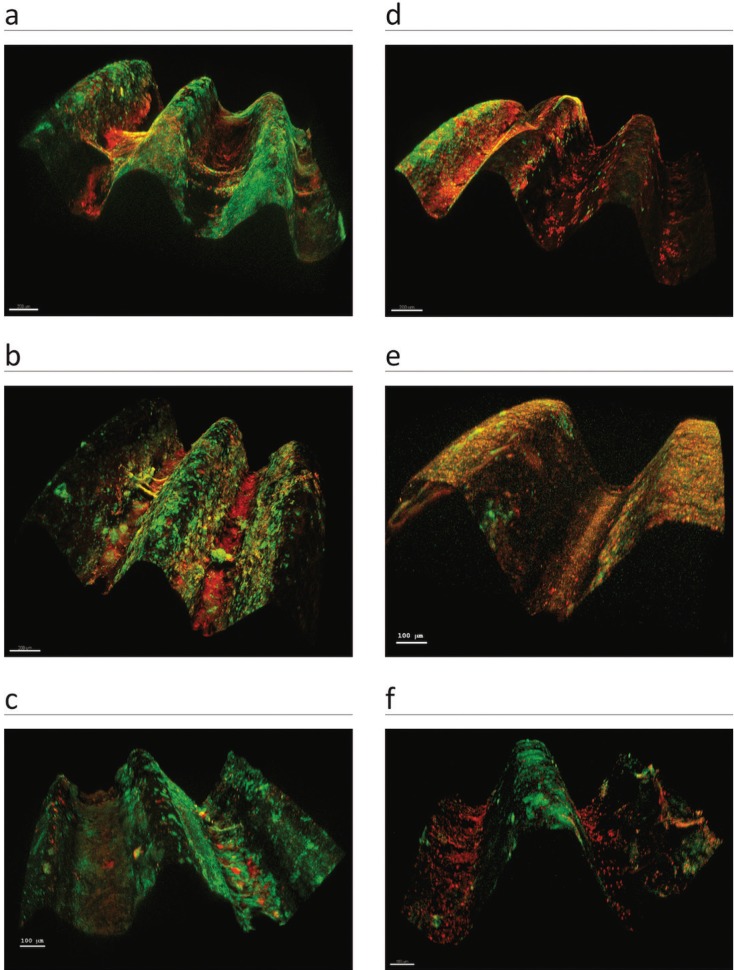
3D reconstructions of stack images captured by CLSM, created using Imaris software. (a-c) Three images of the supragingival area of 3 abutments. Biofilms, mostly colonized by living bacteria (marked in green), cover the majority of the surface. (d-f) Three subgingival zones. Biofilms seem to be less extensive and dead bacteria (marked in red) appear to constitute most of the biofilm.

Regarding the vitality of the biofilm, live cells significantly outnumbered dead ones supragingivally (*p*= 0.005) but not subgingivally (*p*= 0.203) (Fig. 2.2b). The percentage of live cells found supragingivally (51.12%) was greater than the percentage found on the subgingival part of the abutment (19.95%, *p*= 0.017). Also, the percentage of dead cells was greater supragingivally (25.86%) than subgingivally (19.22%, *p*= 0.022) (Fig. 3). The ratio of live to dead bacteria was similar in the supragingival and subgingival portions of the abutment (1.97 vs. 1.41 ; *p*=0.074).

No significant differences in biofilm-covered area were found between the buccal and palatal/lingual areas. Likewise, periodontally-healthy participants and patients with a history of periodontitis had similar outcomes.

Oral biofilms colonizing the entire surface of the abutments were also observed through SEM in the last 10 abutments. The supragingival portion showed higher colonization, whereas epithelial cells were a common find in the subgingival area (Fig. 2.1d, Fig. 2.1e).

## Discussion

Biofilm formation over implants or prosthetic components can play a significant role in the occurrence and progression of peri-implant diseases, just as biofilm formation on teeth is a risk factor for periodontal diseases (24,25). However, some important differences between periodontitis and peri-implantitis have been described. The biofilm formed around teeth has been well studied, especially its growth pattern and 3D structure. Nevertheless, the information available in the literature regarding the characteristics of biofilms formed over exposed implants is very scarce.

Mombelli *et al.* (26) described the microbiota associated with peri-implantitis and pointed out that most studies use techniques that destroy the three-dimensional architecture of the biofilms formed over dental implants (curettes or paper points). Therefore, a removable abutment mimicking a dental implant that allows biofilms to be collected without disruption may be of great interest for research purposes (Fig. 1).

Some authors have employed splints with discs made of titanium (or other materials) to collect biofilm (15,27,28). However, this method does not reproduce a real situation, since the disks are not placed partially in a peri-implant sulcus. Indeed, the soft tissue may act as a protective factor (29). Some authors have studied in vivo biofilms using abutments with different surfaces (9,20,21). However, these abutments were either machined or had a roughness of no more than 0.9 μm. Moreover, their macroscopic appearance was quite different from that of a dental implant. The abutment employed in the present study had a roughness of 1.4-1.5 μm (similar to a commercial dental implant) and included threads which were similar to those of many implant systems. This experimental model made it possible to perform a 3D analysis of an intact 14-day-old in vivo biofilm formed over an exposed dental implant. Thus, this model can be used in future to assess the efficacy of different biofilm removal methods.

The subgingival and supragingival areas showed different results in the present study. This can be explained by the assumption that keratinized mucosa surrounding healthy implants has the potential to prevent subgingival biofilm formation. Even so, approximately 20% of the subgingival portion of the abutments was covered by biofilm ( Table 1). Elter *et al.* (20,21) studied the presence of a 14-day-old biofilm comparing the supragingival coverage area (17.3 ± 23.1%) with the subgingival area (0.8 ± 1.0%) on different abutments. Heuer *et al.* (9) presented similar data, with a coverage of 17.5 ± 18.3% in the supragingival area and 0.8 ± 1.0% of subgingival coverage. Other authors (30) even found a subgingival portion which was completely free of biofilm and colonized with epithelial cells. These studies seem to indicate that keratinized mucosa might be a good barrier when in contact with a smooth surface, avoiding the spread of microorganisms down into the subgingival area. Conversely, rough surfaces seem to be easily colonized by bacteria and this may explain why peri-implantitis can progress rapidly in exposed implants (after bone loss or bone remodeling processes). In these situations, bacteria will easily attach to the implant surface and the keratinized mucosa will not be able to avoid it. Our SEM images confirm this statement. In the present authors’ opinion, the marked differences between the findings of the above-mentioned papers and the outcomes of the present study can be explained by the macro- and microstructure of the experimental abutment that simulates the real situation of an exposed dental implant.

Regarding the vitality of the biofilms analyzed, the live/dead bacteria ratio was slightly higher in the supragingival area than in the subgingival zone. The supragingival locations receive nutrients from the diet, which allow the biofilm to grow easily, whereas the biofilm located on healthy subgingival locations receives a restricted amount of nutrients and oxygen. Since no previous studies have reported this finding, further research is needed to confirm these outcomes.

The strict inclusion/exclusion criteria and the study method (especially the number of visits required and the instructions to avoid oral hygiene measures) made patient enrollment quite difficult. This issue (small sample size) might be considered a limitation even though many studies have included a similar number of participants (9,20,21).

The clinical relevance of the present study is that after 14 days of exposure, the rough surface of a dental implant will be extensively covered by a mature biofilm (38% of the supragingival area and the 21% of the subgingival area). Therefore, the treatment of exposed dental implants should probably include a modification of the surface in order to reduce this fast, widespread colonization.

The proposed in vivo model could be very useful in future to compare the structure of biofilms formed over healthy and diseased implants, and to assess the efficacy of the different decontamination treatments that have been reported in the literature.

In conclusion, the use of removable experimental abutments mimicking exposed dental implants makes it possible to recover undisturbed biofilm that can be analyzed with both CLSM and SEM. When hygiene measures are absent, extensive plaque growth can be observed on these abutments after 14 days. Biofilm coverage was significantly greater in the supragingival zone than in the subgingival portion.
